# Ecological stoichiometry between leaves, litter and soil of dominant species in the forest community of rock-stream periglacial landforms in Mt. Laotudingzi

**DOI:** 10.1371/journal.pone.0328983

**Published:** 2025-08-26

**Authors:** Lan Wang, Daqi Liu

**Affiliations:** School of Geography, Liaoning Normal University, Dalian, China; Western Carolina University, UNITED STATES OF AMERICA

## Abstract

This study investigates the ecological stoichiometric characteristics of carbon (C), nitrogen (N), and phosphorus (P) across the leaf-litter-soil continuum in the block stream forest community of Laotudingzi Mountain, a representative paleo-periglacial landform in northeastern China. Utilizing X-ray fluorescence spectroscopy (XRF), we analyzed 13 dominant tree species (10 broadleaf, 3 coniferous) to unravel nutrient limitation mechanisms and cross-media coupling in this oligotrophic cryogenic ecosystem. Results indicate that P is the primary limiting nutrient, with mean N: P ratios in leaves (12.84), litter (11.25), and soil (8.05) below the global threshold for P limitation (N: P < 14). Cross-media stoichiometric feedbacks reveal efficient P cycling: leaf N: P ratios show significant positive correlations with litter P content (R = 0.557, P < 0.05) and negative correlations with litter C: P ratios (R = −0.581, P < 0.05), while synchronized P dynamics between litter and soil (R = 0.538, P < 0.1) underscore litter decomposition as the primary driver of soil P replenishment. Adaptive nutrient strategies emerge through stoichiometric plasticity, where accelerated P mineralization via low C: P ratios (342.88) compensates for C-N cycling inefficiencies despite inhibitory litter C: N ratios (32.3). These findings highlight the biogeochemical resilience of periglacial forests and provide critical insights for mitigating P limitation, guiding species selection in restoration, and optimizing litter management to enhance ecosystem stability under climate change.

## 1 Introduction

Ecological stoichiometry, as an interdisciplinary framework for deciphering elemental cycling and energy fluxes in ecosystems, focuses on the biogeochemical coupling mechanisms of C, N, and P [1,2]. N and P, as the key limiting elements in terrestrial ecosystems, regulate plant photosynthesis, metabolic pathways, and community succession through synergistic or antagonistic interactions [3]. In forest ecosystems, C, N, and P form dynamic cycling chains via the “plant-litter-soil” continuum: plants fix C through photosynthesis; litter acts as the primary vector for nutrient return, reintroducing C, N, and P into soil via decomposition; while soil physicochemical properties inversely modulate plant nutrient uptake efficiency [4–6]. This cycling process is governed by multi-scale controls including temperature, humidity, and microbial activity, with its underlying mechanisms remaining a central focus of ecological research [7,8].

Ecological stoichiometry is a science that studies the energy balance and multiple chemical element balance of biological systems. It provides a comprehensive method for studying the coupling relationship of C, N, P and other elements in ecosystem processes [9]. N and P are the main limiting elements of natural terrestrial ecosystems. They play an important role in various physiological and metabolic activities during plant growth. They are independent of each other and influence each other, and affect the C fixation of plant leaves. The C, N, and P cycles within the ecosystem are transformed between plants, litter, and soil [10]. The study on the ecological stoichiometric characteristics of plant-litter-soil in forest ecosystems is of great theoretical and practical significance. Litter is a reservoir of organic C and N in forest ecosystems, and it is also a hub for material exchange between soil and plants [11]. Litter plays an important role in the storage of organic matter and nutrient cycling in forest ecosystems, and is one of the natural sources of forest soil fertility. The accumulation of soil organic matter and nutrients mainly comes from the return of various forms of litter [12]. Plants fix C through photosynthesis, transfer part of C to the soil, and gradually compensate C and nutrients to the soil in the form of litter [13]. The changes of soil nutrient supply, plant nutrient demand, self-regulation of plant nutrient demand and nutrient return during litter decomposition affect each other [14], which makes the study of nutrient content in ‘ plant-litter-soil ‘ system very complicated [13]. As an ‘ ecological stoichiometry ‘ for studying the energy and multi-element balance of biological systems, provides an effective means to reveal the stoichiometric relationships and laws of C, N, P and other elements in the ‘ plant-litter-soil ‘ ecological process [14]. There have been many studies on the general characteristics of surface litter, plant or soil, but most of these studies take any one or both of litter, plant or soil as the research object, and the differences in the ecological stoichiometric characteristics of C, N and P in the ‘ plant-litter-soil ‘ continuum and their internal correlations are still insufficient. The rock physical weathering process of the ancient periglacial landform in the mountainous area of eastern Liaoning leads to the slow rate of soil formation and the formation of a thin layer of coarse soil. Its low P availability constitutes a key geographical limiting factor for vegetation growth. Therefore, this paper chooses several dominant tree species in the ancient periglacial landform of Laotudingzi as the research object, and studies the relationship between litter and soil, which has important practical significance for revealing the interaction and balance relationship between C, N and P.

## 2 Study area

**Fig 1:**
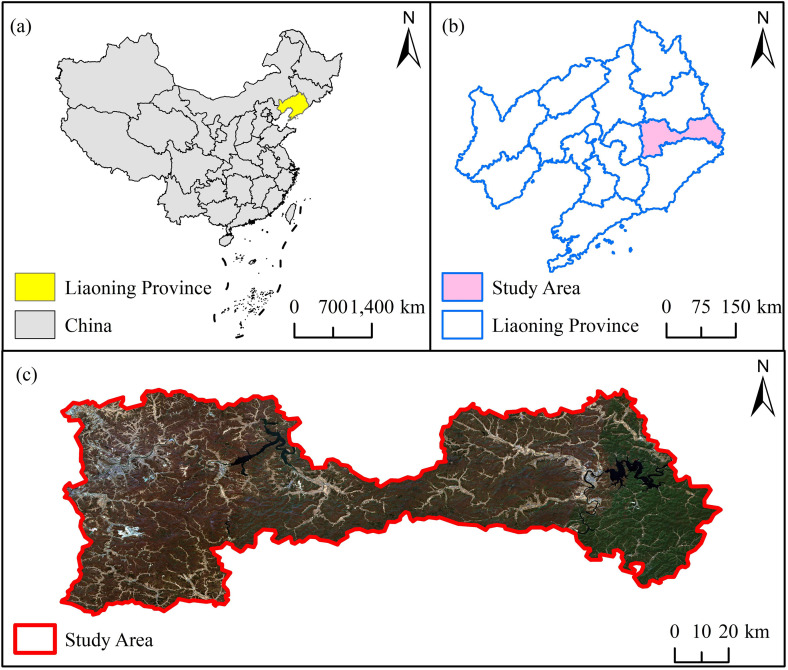
Map of study area. Shows the geographical location and topography of Laotudingzi Mountain.

Laotudingzi National Nature Reserve, situated at the administrative boundary between Huanren Manchu Autonomous County (Benxi City) and Xinbin Manchu Autonomous County (Fushun City) in Liaoning Province, spans geographic coordinates 124°41′13″E–125°5′15″E and 41°11′11″N–41°21′34″N, with a total area of 152.17 km^2^ (Fig 1). The Laotudingzi paleo-periglacial area, with its main peak at 1,367.3 m a.s.l. and a relative relief of 867 m, constitutes a unique geomorphic unit in northeastern China. Characterized by a mid-temperate montane forest ecosystem, it sustains a forest coverage rate of 97%. The main peak and surrounding ridges are dotted with periglacial landforms spanning approximately 1,500 hectares [15]. Brown soils and dark brown soils alternate across the area, showing marked vertical zonation in soil thickness (summit: < 30 cm; valley slopes: 30–60 cm). This region preserves transitional floristic characteristics between the Changbai Mountains and North China flora, including relict species such as *Neottianthe cucullata* [11,12]. The area exhibits pronounced vertical vegetation zonation: from 600 m a.s.l. to the summit, deciduous broadleaf forests, coniferous forests, mixed coniferous-broadleaf forests, dark coniferous forests, and dwarf Betula ermanii scrub communities form altitudinal sequences. Surface lithology features chaotic boulder accumulations, alongside retarded pedogenesis and localized soil horizon truncation, resulting in constrained vegetation succession and ecosystem fragility [10,16].

Climatologically classified as temperate continental monsoon, the reserve has a mean annual temperature of 6°C, annual precipitation of 827.8 mm (73% relative humidity), extreme temperatures ranging from 38°C to −33.2°C, and a frost-free period of 139 days. Enhanced orographic lifting by Pacific southeast monsoons drives pronounced summer rain-hot season coupling, yet high surface runoff coefficients (70%) and flashy hydrological responses characterize slope processes [11,17]. Soil profiles dominated by brown soils and mountain dark brown soils (30–60 cm thickness) exhibit loose structures and high organic matter content, classified into 2 soil orders, 3 suborders, and 7 soil families, providing essential substrates for forest development. The region harbors exceptional biodiversity hotspots, including relict gymnosperms (*Taxus chinensis, Magnolia sieboldii*) and the globally endemic orchid *Neottianthe cucullata*. Its flora demonstrates transitional characteristics between the southwestern margin of the Changbai Mountain floristic region and the North China floristic region [16,10].

## 3 Sampling and analysis

**Fig 2:**
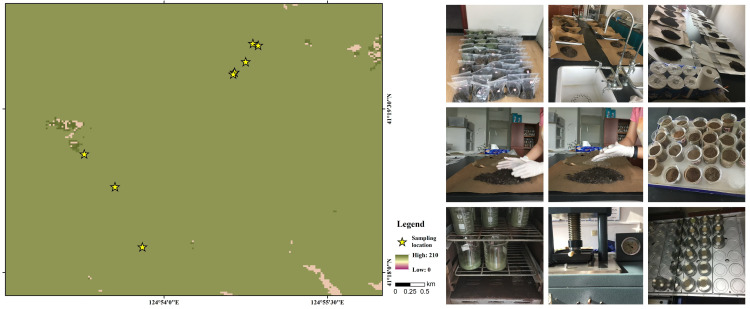
Sample collection and treatment. The location of sampling points and laboratory process.

Based on prior biogeographical investigations of the Changbai Mountains-North China floristic transition zone [10,18], 13 dominant tree species were identified in the Laotudingzi paleo-block stream forest community, including 10 broadleaf species (*Acer pseudo-Sieboldianum, Carpinus cordata, Tilia amurensis, Acer mono Maxim, Ulmus laciniata, Fraxinus mandschurica, Quercus mongolica, Acer barbinerve, Prunus serrulata, Betula ermanii) and 3 coniferous species (Abies nephrolepis, Pinus koraiensis, Abies holophylla*). Sampling was conducted in periglacial landform-concentrated areas (Dongpo Changzigou and Beipo Sandaogou valleys) using a stratified random approach. For each species, geospatial coordinates (WGS84), tree height, diameter at breast height, and crown architecture were recorded (Table 1). Foliage samples (4–8 canopy branches per tree) were stored in hermetic polyethylene bags for natural air-drying. Litter and soil samples were collected via quadrat harvesting method beneath each tree and within the sampling quadrats, respectively. The soil samples were collected by plum blossom sampling method, and 5 sub-samples were collected in a 3m × 3m grid. The impurities such as gravel were removed, and 1.0 kg of mixed samples were left in a polyethylene self-sealing bag and labeled. The collected samples were placed in a cool and ventilated place for natural air drying. After drying, they were ground through a 100-mesh nylon sieve, and 150 g of the particle size sample under the sieve was weighed by the quartering method. A total of 13 foliar, 22 litter (shared among clustered *Acer pseudo-Sieboldianum*, *Carpinus cordata*, and *Tilia amurensis*), and 22 soil specimens were processed. Laboratory procedures included oven-drying at 65°C, pulverization using an SM-1 vibratory ball mill (Dandong Yuan Instrument Co., Ltd.), pellet preparation via BP-1 press, and elemental (C, N, P) quantification using a Rigaku ZSX Primus II wavelength-dispersive X-ray fluorescence spectrometer. Soil samples were sieved (<2 mm) and processed identically. Pearson correlations and ANOVA for foliar-litter-soil elemental relationships were performed using IBM SPSS Statistics 26 and Origin 2022, with spatial stoichiometric patterns illustrated in Fig 2.

**Table 1:** Morphometric and Geospatial Data of Dominant Tree Species.

Dominant Species	Geospatial Coordinates			Morphometric Parameters		Associated Flora
	Latitude(N)	Longitude(E)	Elevation(m)	DBH(cm)	Age(a)	
*Acer pseudo-Sieboldianum*	41°19′50″	124°54′39″	761	18	30	*Carpinus cordata, Tilia amurensis*
*Carpinus cordata*	41°19′50″	124°54′39″	761	12	50	*Acer pseudo-Sieboldianum, Tilia amurensis*
*Tilia amurensis*	41°19′50″	124°54′39″	761	24	70	*Acer pseudo-Sieboldianum, Carpinus cordata*
*Acer mono Maxim*	41°19′49″	124°54′38″	728	8	18	*Carpinus cordata, Sorbus alnifolia, Actinidia kolomikta*
*Ulmus laciniata*	41°19′56″	124°54′45″	687	4	10	*Acer mono Maxim, Acer pseudosieboldianum, Pinus koraiensis Siebold, Corylus heterophylla*
*Fraxinus mandschurica*	41°20′05″	124°54′52″	629	30	60	*Spiraea salicifolia, Sambucus williamsii*
*Quercus mongolica*	41°18′14″	124°53′48″	882	20	40	*Fraxinus chinensis*
*Acer barbinerve*	41°18′47″	124°53′33″	1107	14	30	*Fraxinus mandshurica, Sambucus williamsii*
*Prunus serrulata*	41°18′47″	124°53′33″	1109	18	30	*Quercus mongolica, Acer ukurunduense, Acer pseudosieboldianum*
*Betula ermanii.*	41°19′05″	124°53′16″	1255	10	70	*Acer pictum Thunb.*
*Abies nephrolepis*	41°18′47″	124°53′33″	1109	40	110	*Cerasus maximowiczii*
*Pinus koraiensis*	41°20′06″	124°54′49″	641	18	40	*Acer mono Maxim.*
*Abies holophylla*	41°20′05″	124°54′52″	616	45	90	*Spiraea salicifolia, Sambucus williamsii, Autumn pear*

This table lists the 13 dominant tree species in the Laotudingzi forest community, including their geospatial coordinates (latitude in °N, longitude in °E, elevation in m), morphometric parameters (diameter at breast height in cm, age in years), and associated flora. It provides a comprehensive overview of species-specific characteristics to support ecological analysis.

## 4 Results and analysis

### 4.1 Ecological stoichiometric characteristics of dominant tree species in Laotudingzi paleo-block stream forest

**Fig 3:**
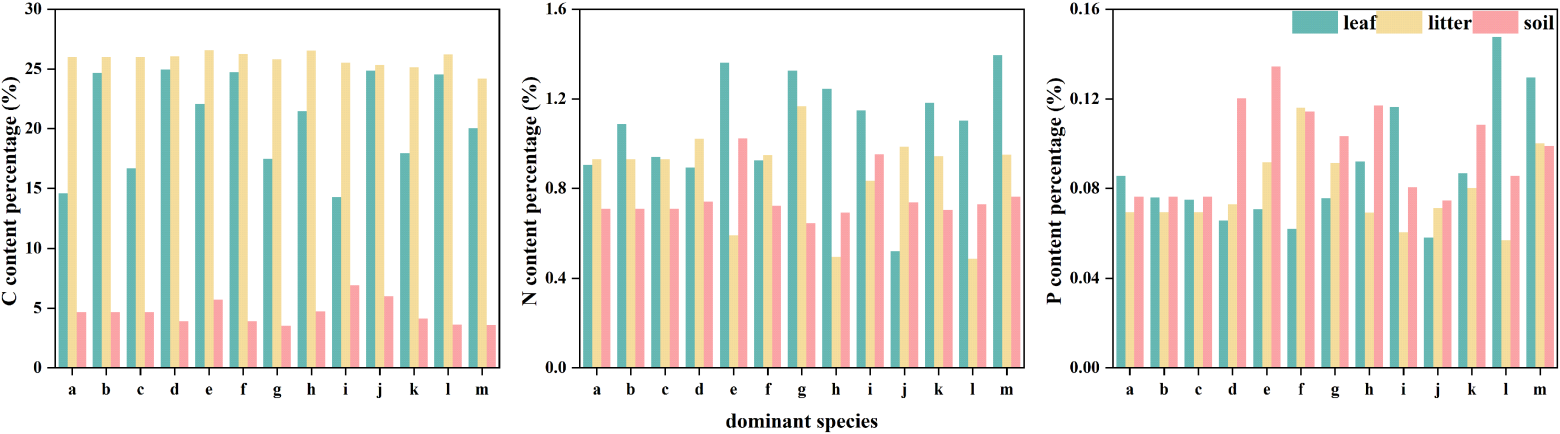
The contents of C, N and P in leaves, litter and soil of dominant tree species. Displays bar charts or graphs comparing the concentrations of C, N, and P across leaves, litter, and soil components for the 13 dominant tree species.

The leaf C content of 13 dominant tree species in the Gushihe forest community ranged from 14.27% to 24.95% (Table 2), with an average of 20.65%, and the coefficient of variation (C.V.) was 19.67%. Moderate variability (C.V. ≤ 15% is defined as slight variability; 15% < C.V. ≤ 35% was moderate variation; 35% < C.V. ≤ 100% was highly variable. C.V. ≥ 100% is strong variation [19]). Among them, the C content of *Acer mono Maxim*, *Prunus serrulata*, *Pinus koraiensis*, and other 8 tree species was higher than the average, reflecting higher C assimilation capacity; five species such as *Betula ermanii* and *Quercus mongolica* were lower than the mean (Fig 3). The content of C element in the litter under the tree ranged from 24.20% to 26.58%, with an average of about 25.83%, which was a slight variation. The content of C in the soil under the tree ranged from 3.51% to 6.94% (Table 2), with an average of about 4.63%, which belonged to moderate variation.

**Table 2:** Statistical characteristics of leaf, litter and soil ecological stoichiometry of dominant tree species.

Parameter(%)		C	N	P	C:N	C:P	N:P
Mean	leaf	20.65	1.08	0.09	20.75	257.76	12.84
	litter	25.83	0.86	0.08	32.31	342.88	11.25
	soil	4.63	0.76	0.1	6.11	50.17	8.05
Content Range	leaf	14.27-24.95	0.52-1.40	0.06-0.15	12.44-47.67	122.49-427.57	7.46-19.26
	litter	24.20-26.58	0.49-1.17	0.06-0.12	22.13-53.90	225.91-461.27	6.43-14.01
	soil	3.51-6.94	0.65-1.02	0.08-0.14	4.73-8.13	32.69-86.05	5.91-11.83
CV(%)	leaf	19.67	22.42	31.11	45.71	39	26.01
	litter	2.507	24.09	21.6	33.77	20.01	25.2
	soil	22.42	14.12	20.97	16.28	36.14	22.58
*Acer pseudo-Sieboldianum*	leaf	14.62	0.91	0.09	16.14	170.62	10.57
	litter	26	0.93	0.07	27.97	374.4	13.39
	soil	4.69	0.71	0.08	6.59	61.42	9.31
*Carpinus cordata*	leaf	24.67	1.09	0.08	22.7	324.7	14.31
	litter	26	0.93	0.07	27.97	374.4	13.39
	soil	4.69	0.71	0.08	6.59	61.42	9.31
*Tilia amurensis*	leaf	16.67	0.94	0.07	17.71	222.28	12.55
	litter	26	0.93	0.07	27.97	374.4	13.39
	soil	4.69	0.71	0.08	6.59	61.42	9.31
*Acer mono Maxim*	leaf	24.95	0.89	0.07	27.94	379.63	13.59
	litter	26.09	1.02	0.07	25.54	357.73	14.01
	soil	3.93	0.74	0.12	5.3	32.69	6.17
*Ulmus laciniata*	leaf	22.07	1.36	0.07	16.2	312.03	19.26
	litter	26.58	0.59	0.09	44.87	289.98	6.46
	soil	5.72	1.02	0.13	5.59	42.52	7.6
*Fraxinus mandschurica*	leaf	24.75	0.93	0.06	26.74	399.45	14.94
	litter	26.25	0.95	0.12	27.67	225.91	8.17
	soil	3.92	0.72	0.11	5.42	34.34	6.33
*Quercus mongolica*	leaf	17.49	1.33	0.08	13.19	231.27	17.53
	litter	25.84	1.17	0.09	22.13	282.73	12.78
	soil	3.51	0.65	0.1	5.43	34.01	6.26
*Acer barbinerve*	leaf	21.46	1.25	0.09	17.23	233.08	13.53
	litter	26.55	0.5	0.07	53.55	383.59	7.16
	soil	4.75	0.69	0.12	6.87	40.59	5.91
*Prunus serrulata*	leaf	14.27	1.15	0.12	12.44	122.49	9.84
	litter	25.55	0.83	0.06	30.63	422.08	13.78
	soil	6.94	0.95	0.08	7.28	86.05	11.83
*Betula ermanii.*	leaf	24.87	0.52	0.06	47.67	427.57	8.97
	litter	25.34	0.99	0.07	25.72	355.52	13.82
	soil	6.01	0.74	0.07	8.13	80.56	9.91
*Abies nephrolepis*	leaf	17.95	1.18	0.09	15.18	206.84	13.63
	litter	25.14	0.94	0.08	26.64	313.75	11.78
	soil	4.16	0.7	0.11	5.91	38.42	6.5
*Pinus koraiensis*	leaf	24.56	1.1	0.15	22.29	166.33	7.46
	litter	26.23	0.49	0.06	53.89	461.26	8.56
	soil	3.62	0.73	0.09	4.96	42.21	8.51
*Abies holophylla*	leaf	20.04	1.4	0.13	14.36	154.61	10.77
	litter	24.19	0.95	0.1	25.43	241.67	9.5
	soil	3.61	0.76	0.1	4.73	36.53	7.73

This table summarizes mean values, content ranges, and coefficient of variation (CV in %) for carbon (C), nitrogen (N), phosphorus (P) content (in %), and their ratios (C:N, C:P, N:P) across leaf, litter, and soil samples of dominant tree species. It highlights spatial variability and nutrient dynamics to identify biogeochemical patterns in the ecosystem.

The leaf N content ranged from 0.52% to 1.40%, with an average of 1.08%, which was significantly lower than the reference value of coniferous and broad-leaved mixed forest in Changbai Mountain (2.03%) [20], and C.V.22.42% was moderate variation. The contents of 8 kinds of N such as *Fraxinus mandschurica* and *Ulmus laciniata* were higher, and the contents of 5 kinds of N such as *Tilia amurensis* and *Pinus koraiensis* were lower. The content of N element in the litter under the tree ranged from 0.49% to 1.17%, with an average value of about 0.86%, and the standard deviation rate was 24.09%, which was moderate variation. Among them, the N content of litter under *Abies holophylla*, *Acer barbinerve* and *Ulmus laciniata* was significantly lower than the average level, and there was still a certain degree of opposite relationship between the order of N content and C order. The soil N content ranged from 0.65% to 1.02%, with an average of 0.76%, and the standard deviation was 14.12%, which was a slight variation. Among them, the soil N content of *Abies holophylla, Acer barbinerve* and *Ulmus laciniata* was significantly higher than the average level, and there was still a certain degree of similarity between the N element content ranking and the C ranking.

The leaf P content ranged from 0.06% to 0.15%, with an average of 0.09%. Only four species such as *Abies holophylla* and *Fraxinus mandschurica* were higher than the average, and C.V.31.11% reflected moderate spatial heterogeneity, which was significantly lower than the reference value of Changbai Mountain (0.166%) [20]. The content of P element in the litter under the trees ranged from 0.06% to 0.12%, with an average value of about 0.08%. Among them, *Pinus koraiensis, Fraxinus mandschurica, Ulmus laciniata*, *Quercus mongolica* and *Betula ermanii* were higher than the average content level in turn, and the standard deviation rate was 21.60%, which was still moderate variation. From the element content and variation level of the litter under the tree, it can be seen that the plant has a good reabsorption capacity for litter nutrient elements. The content of P element in soil ranged from 0.08% to 0.14%, with an average of 0.10%. Among them, *Ulmus laciniata, Acer mono Maxim, Acer barbinerve, Pinus koraiensis, Betula ermanii, Quercus mongolica* and *Fraxinus mandschurica* were higher than the average content level in turn, with a standard deviation rate of 20.97%, which was still moderate variation.

The ecological stoichiometric ratio showed that the leaf C: N of the dominant species in the ancient stone river of Laotudingzi ranged from 12.44 to 47.67 (Table 2), with an average value of 20.75 (lower than the ratio of 24.7 of the same order in Changbai Mountain [20]), and the standard deviation rate was 45.71, which belonged to high variation. Among them, the C: N of *Betula ermanii*, *Acer mono Maxim*, *Fraxinus mandschurica*, *Carpinus cordata* and *Pinus koraiensis* were higher than the average level. The C: N of litter layer ranged from 22.13 to 53.90, with an average of 32.31 and a standard deviation of 33.77, which belonged to moderate variation. Among them, the C: N ratio of litter decomposition layer under *Pinus koraiensis* and *Acer barbinerve* was more than 50. The C: N ratio in the soil layer under the trees ranged from 4.73 to 8.13, with an average of 6.11 and a standard deviation of 16.28, which was moderate variation.

Leaf C: P ranged from 122.49 to 427.57, with an average of 257.76. The order of C: P of different dominant tree species from high to low was: *Betula ermanii* > *Fraxinus mandshurica* > *Acer pictum* Thunb > *Carpinus cordata* > *Ulmus laciniata* > *Acer barbinerve* > *Quercus mongolica* > *Tilia amurensis* > *Betula ermanii* > *Acer pseudo-Sieboldianum* > *Pinus koraiensis* > *Abies holophylla* > *Prunus serrulata*. The standard deviation is 39.00, which is a high degree of variation. Litter C: P ranged from 225.91 to 461.27, with an average of 342.88. Litter C: P ranged from 32.69 to 86.05, with an average of 50.17.

Leaf N: P ranged from 7.46 to 19.26, with an average value of 12.84, which was slightly lower than the average level of 321 and 13 in the adjacent Changbai Mountains [20], and also lower than the average level of forest communities in eastern China (313.9) [21], reflecting that the absorption efficiency of P by Gushihe vegetation in Laotudingzi was higher, and plant growth was mostly affected by N and P elements. The order of N: P of different dominant tree species from high to low was: *Ulmus laciniata* > *Quercus mongolica* > *Fraxinus mandschurica* > *Carpinus cordata* > *Abies nephrolepis* > *Acer mono Maxim* > *Acer barbinerve* > *Tilia amurensis* > *Abies holophylla* > *Acer pseudo-Sieboldianum* > *Prunus serrulata* > *Betula ermanii*> *Pinus koraiensis*. The standard deviation rate was 26.01, which belonged to moderate variation. It reflects that the nutrient acquisition of plants has good stability, and there are some differences in the ratio of the three elements among different dominant tree species. Litter N: P ranged from 6.46 to 14.01, with an average of 11.25. Soil N: P ranged from 5.91 to 11.83, with an average of 8.05.

### 4.2 Ecological stoichiometric relationships between foliar and litter in Laotudingzi paleo-block stream forest

**Fig 4:**
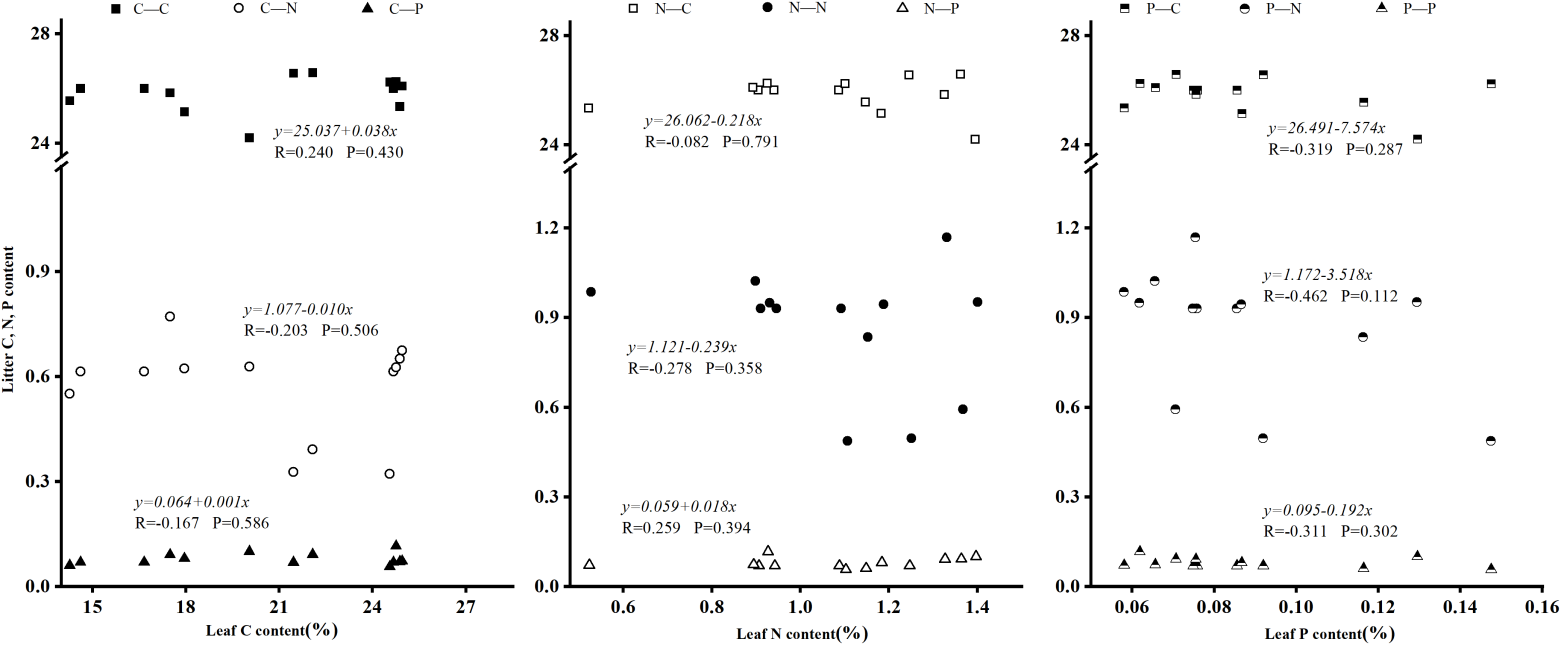
Relationship between leaf element content and litter element content of dominant species. Presents a scatter plot or diagram demonstrating correlations between elemental concentrations (C, N, P) in leaves and litter for dominant species, based on statistical analyses like Pearson correlation.

**Fig 5:**
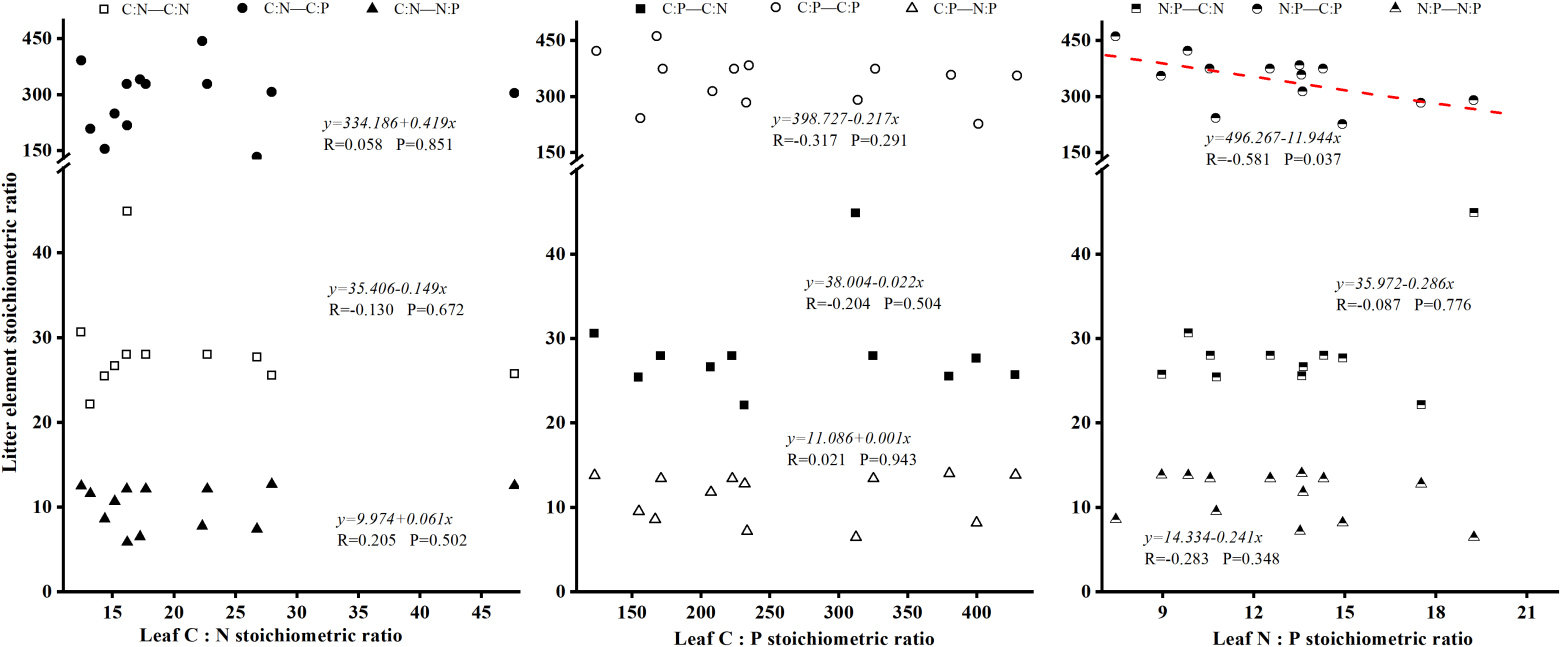
Relationship between leaf element stoichiometry of dominant species and litter element stoichiometry. Features a scatter plot showing how stoichiometric ratios (e.g., C:N, C:P, N:P) in leaves correlate with those in litter, emphasizing trends such as significant negative correlations.

Correlation analysis between foliar C, N, P content (and ratios) and litter elemental composition revealed no statistically significant correlations between foliar and litter elemental concentrations (Fig 4). There was also a significant negative correlation between leaf N: P and litter C: P (P < 0.05), and the correlation coefficient was R = −0.581 (Fig 5). The N: P ratio of plant leaves can reflect the N and P nutrient limitation pattern of plant growth process, and the P element of litter under the tree is the limiting element of plants in this area.

### 4.3 Ecological stoichiometric relationships between foliar and soil in Laotudingzi paleo-block stream forest

**Fig 6:**
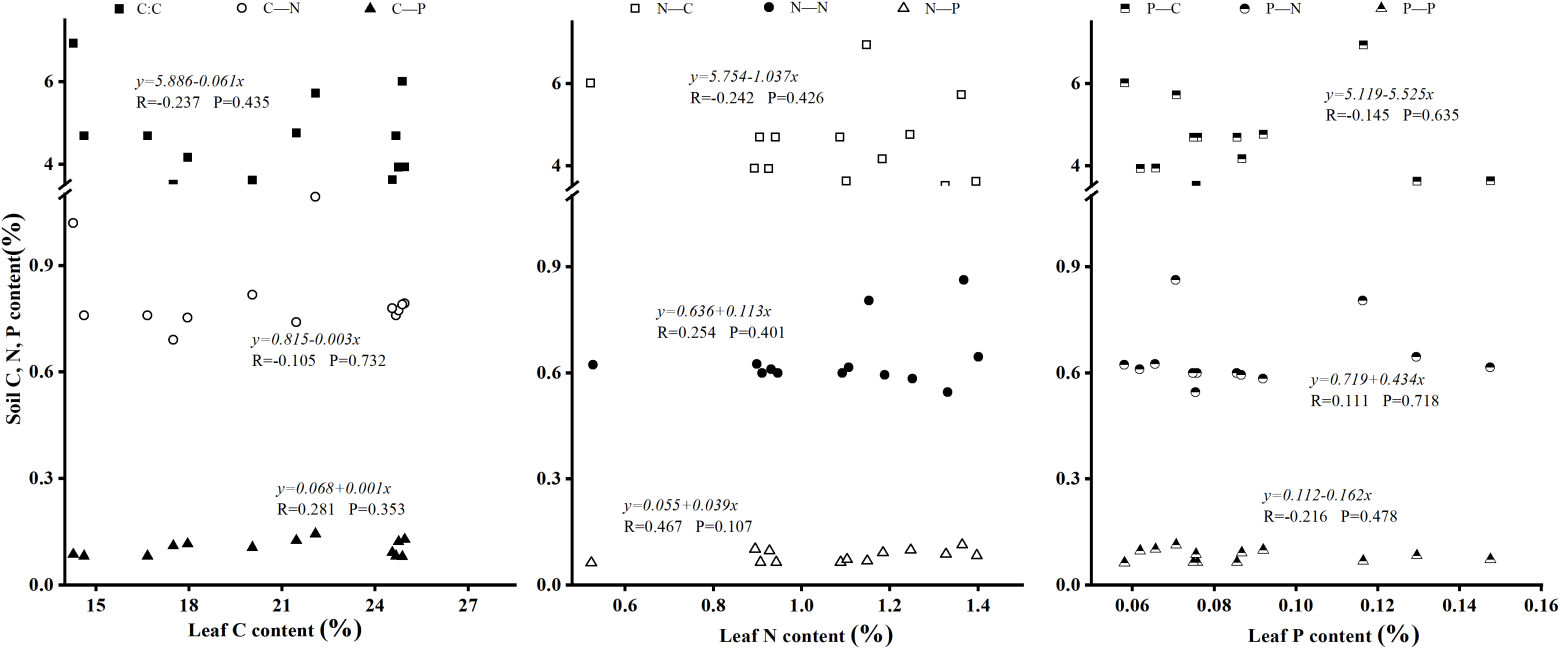
Relationship between leaf element content of dominant species and soil element content. Scatter plot depicting the relationship between leaf element content and soil element content.

**Fig 7:**
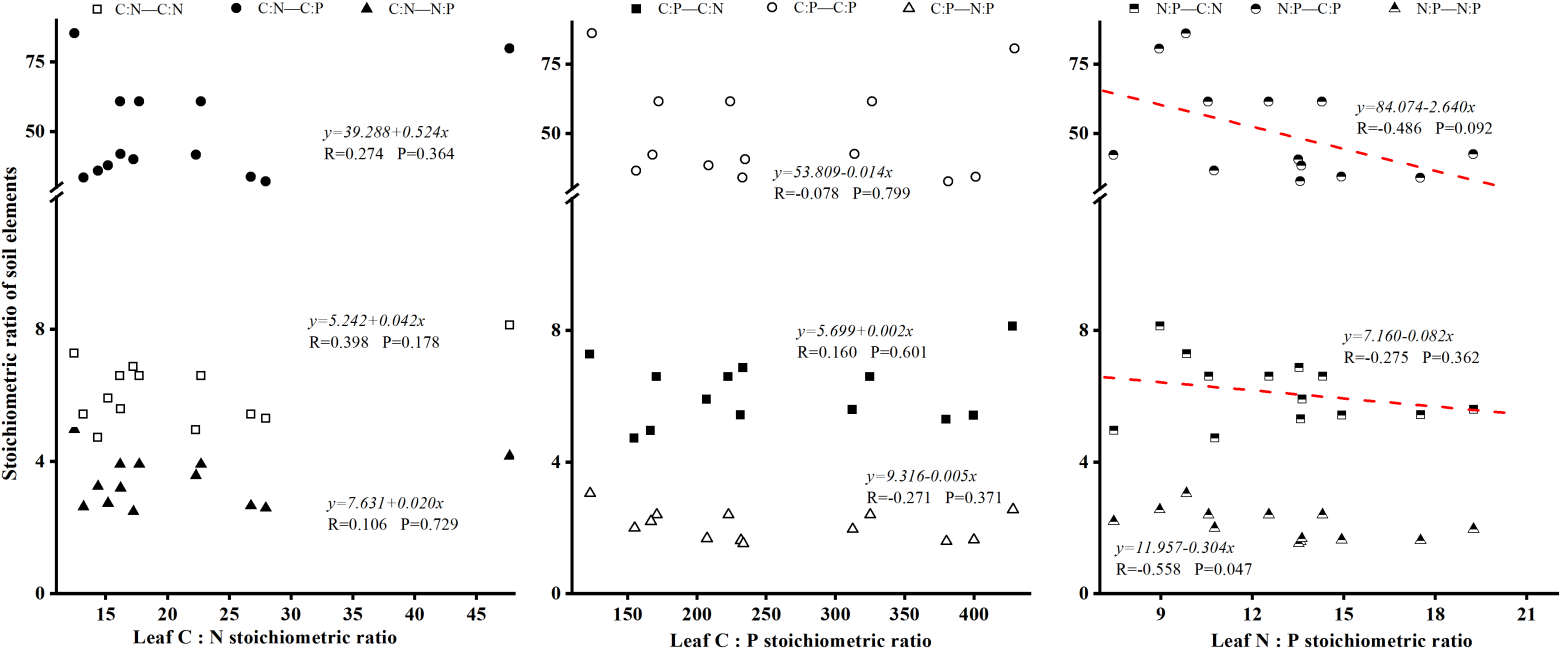
Relationship between leaf element stoichiometry of dominant species and soil element stoichiometry. Shows a scatter plot illustrating how leaf stoichiometric ratios (e.g., N:P) relate to soil ratios (e.g., C:N, C:P), including significant negative correlations identified.

Correlation analysis revealed no statistically significant linear relationships between foliar elemental content and underlying soil elemental composition in the Laotudingzi forest community (Fig 6). There was also a significant negative linear correlation between leaf N: P and soil C: N under the tree (P < 0.05), and the correlation coefficient R = −0.558; there was also a significant negative correlation with soil C: P under the tree (P < 0.1), and the correlation coefficient R = −0.486, the correlation was not strong (Fig 7). The increase of plant N content or N: P ratio can reflect the good growth of plants, while the soil C: N, C: P ratio is small, or the P content is higher, which usually indicates that the nutrient cycle rate of the soil environment is faster. The two promote each other to achieve the stable growth of plants.

### 4.4 Ecological stoichiometric relationships between litter and soil in Laotudingzi paleo-block stream forest

**Fig 8:**
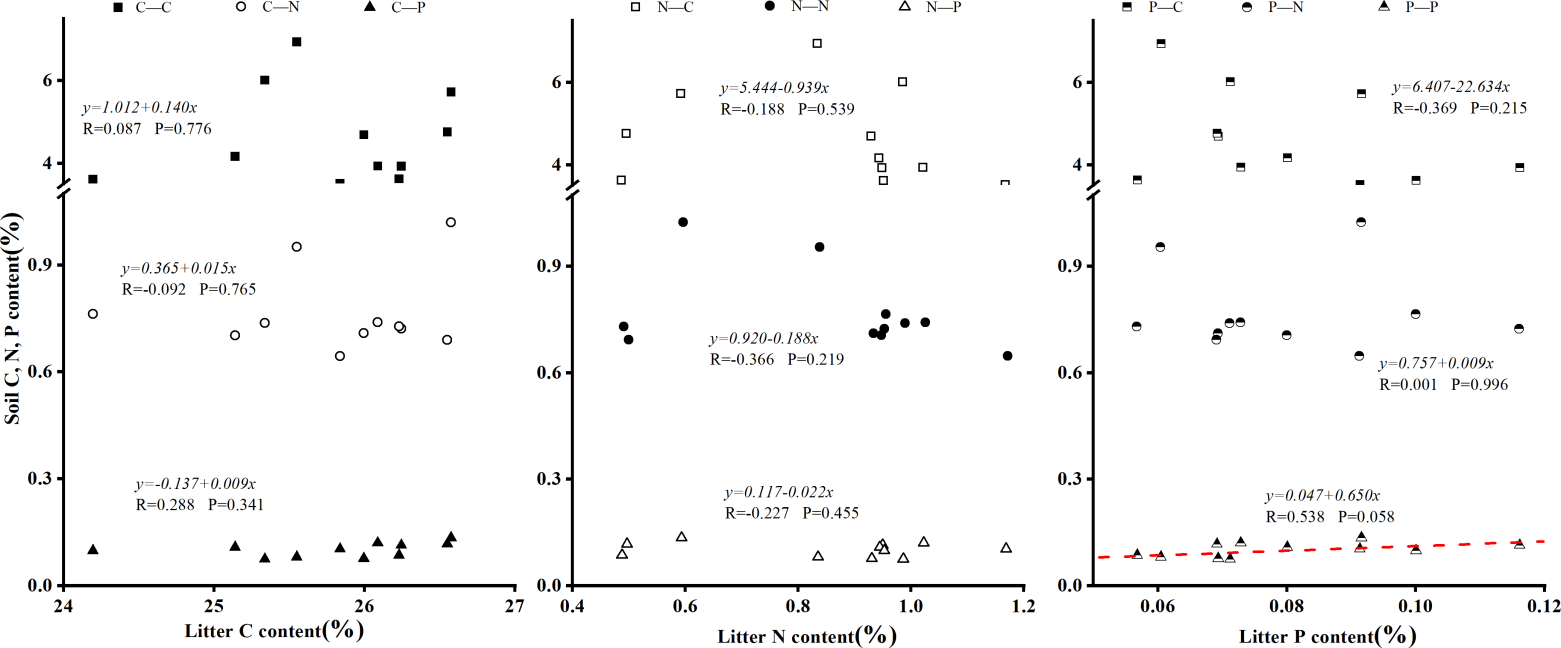
Relationship between litter element content and soil element content. Displays a scatter plot highlighting correlations, particularly for phosphorus (P), between litter and soil elemental concentrations, with significant positive trends observed.

**Fig 9:**
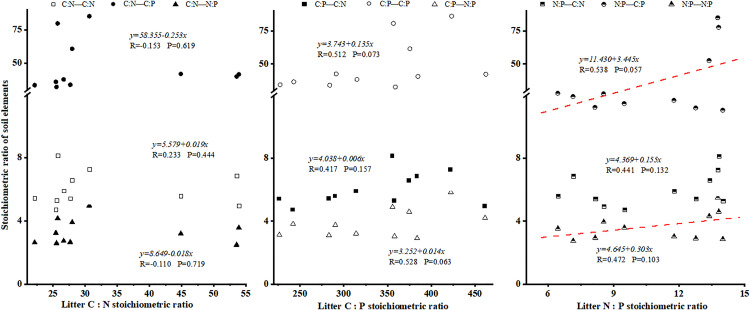
Relationship between litter element stoichiometry and soil element stoichiometry. Features a scatter plot demonstrating how stoichiometric ratios in litter (e.g., N:P) correlate with those in soil (e.g., C:P), showing positive but non-significant trends in the data.

Correlation analysis revealed a significant positive linear correlation between litter P content and soil P content beneath dominant tree species (R = 0.538, P < 0.1; Fig 8). Additionally, litter N: P ratio exhibited a significant positive linear correlation with soil C: P ratio (R = 0.538, P < 0.1; Fig 9). A positive but non-significant trend was observed between litter N: P ratio and soil N: P ratio (R = 0.472, P = 0.103). These findings indicate that P is the limiting element for vegetation growth in this region. Notably, the synchronized dynamics between fully decomposed litter layer (Oa horizon) P content and soil layer (B horizon) P content suggest tight nutrient coupling during biogeochemical cycling, where litter decomposition directly regulates soil P availability through mineralization-immobilization turnover.

## 5 Discussion

This study revealed the biogeochemical cycle characteristics and driving mechanism of C, N and P elements in the forest ecosystem of Laotudingzi ancient periglacial landform in eastern Liaoning. From the perspective of geography, the interaction between the unique periglacial geomorphological environment and the temperate monsoon climate in the region has jointly shaped a special element cycle pattern [22]. The rock debris accumulation of the ancient periglacial landform leads to the slow soil formation process. The P in the granite parent material mainly exists in the form of insoluble secondary phosphate [23], and the runoff coefficient of the slope is as high as 70% [11], resulting in strong leaching of P. This is consistent with the research conclusions of other periglacial regions in the world [24], but the soil P content in this area is significantly lower than that in the above regions [25], reflecting the stronger hydrological erosion effect in the East Asian monsoon region. It is worth noting that the elevation gradient has a nonlinear regulation on the distribution of P: in the periglacial core zone above 1100 m, freeze-thaw promotes the development of rock fissures, but accelerates the chemical weathering of primary minerals [26], which explains the phenomenon of relatively high P content in the soil under the Abies nephrolepis forest. Plants achieve niche complementarity through life form differentiation. Broad-leaved tree species (such as *Fraxinus mandshurica*) adopt the ‘ rapid cycle ‘ strategy, and their leaf N: P is close to the Redfield ratio [27], and the litter P return is significantly higher than that of coniferous trees, which is similar to the element cycle model of low-altitude warm temperate deciduous forests [28]. Coniferous tree species (such as *Pinus koraiensis*) adopt a ‘ conservative storage ‘ strategy to delay decomposition and reduce nutrient loss through high C: N litter, similar to the characteristics of the cold temperate taiga forest [29]. This life form differentiation forms a ‘ nutrient cycling coupling chain ‘ on the vertical band spectrum, which is significantly different from the vertical band structure of vegetation in Changbai Mountain [30], reflecting the marginal effect of the transition from North China flora to Changbai Mountain flora. It is recommended to adopt the ‘ landform-vegetation collaborative restoration ‘ model: deep-rooted tree species (such as *Ulmus pumila*) are preferentially planted in the active zone of the periglacial zone, and their roots can penetrate the debris layer to promote soil formation [31]. The broad-leaved-coniferous mixed forest was constructed on the slope skirt, and the soil structure was improved by using the complementary litter traits [32]. Future research needs to combine stable isotope (δ15N, δ13C) tracer technology [33] to quantify the element migration flux in different geomorphological sites.

These findings have critical implications for managing cold-temperate montane ecosystems under global climate change. The identified P limitation (N: P < 14) suggests that future warming may exacerbate nutrient constraints by accelerating organic matter decomposition while P weathering remains slow. To enhance ecosystem resilience, management strategies should prioritize: For the active periglacial zone, it is recommended to restore deep-rooted species (such as *Ulmus laciniata* and *Fraxinus mandshurica*) to enhance bedrock weathering and debris stabilization, promote soil formation processes, and maintain low C: P litter-driven phosphorus cycle efficiency. At the same time, it is necessary to establish a soil C: N: P stoichiometric ratio monitoring system (trigger warning when N: P < 14) to cope with the risk of nutrient imbalance caused by accelerated decomposition of organic matter and delayed weathering of P caused by warming. Management measures should give priority to protecting the structure of mixed forests, avoiding the destruction of stoichiometric balance by coniferous pure forests with high C: N litter, and alleviating phosphorus loss caused by hydrological erosion by retaining the litter layer in the periglacial zone. In addition, microbial-mediated phosphorus cycling mechanisms (such as phosphatase activity and mycorrhizal networks) need to be integrated to enhance the biogeochemical resilience of ecosystems to climate warming. These strategies together constitute a ‘ weathering-vegetation-microorganism ‘ synergistic regulatory framework, which can effectively alleviate the P limitation of low-temperature forests and enhance landscape stability.

## 6 Conclusions

This study systematically investigated the ecological stoichiometric characteristics of 13 dominant tree species in the Laotudingzi paleo-block stream forest community (Liaodong Mountains, China) through X-ray fluorescence spectroscopy (XRF) and geostatistical analyses. The results revealed distinct biogeochemical patterns across foliar, litter, and soil components.

(1) The N: P ratios (mean values were 12.84, 11.25, and 8.05, respectively) of leaves, litter, and soil in the ancient Shihe forest community were lower than the global P limitation threshold (N: P < 14), confirming that P is the primary limiting factor for the ecosystem, which is closely related to the low P weathering rate caused by the poor matrix of the periglacial landform (granite parent material, low soil layer thickness). Coniferous species reduced nutrient loss by slowing down decomposition through high C: N litter, while broad-leaved species supplemented soil P pool by efficient return of litter P, reflecting the adaptive strategies of different life forms to P limitation.
(2) Soil C: N and C: P ratios significantly affected nutrient dynamics: low C: N soil promoted N mineralization and increased plant N uptake (leaf N content 1.02%), while high C: P soil led plants to adapt to low P environment by increasing N: P (mean 8.05), forming a negative feedback mechanism to maintain system homeostasis.
(3) The positive correlation between litter P and soil P reflected the efficient transfer of P at the decomposition interface, while the negative correlation between leaf N: P and soil N: P was due to the preferential allocation of resources to P acquisition by plants when N was sufficient. The high C: N litter of coniferous tree species inhibited decomposition to adapt to barren habitats, while the broad-leaved tree species increased soil fertility through rapid turnover of litter, and the two synergistically maintained community diversity.
(4) This study has important implications for the ecological restoration of periglacial landforms. It is recommended to give priority to the introduction of tree species with high P use efficiency (such as *Ulmus laciniata*) and promote P cycle through litter management (such as retaining broad-leaved litter). In the future, it is necessary to further reveal the biological driving mechanism of litter decomposition in combination with microbial community analysis.

## Supporting information

S1 Data Data. (XLSX)
